# Supplementary material for: Cooperation and the evolution of hunter-gatherer storytelling
Source: Nat Commun. 2017 Dec 5;8:1853. doi: 10.1038/s41467-017-02036-8 (PMC5717173; doi:10.1038/s41467-017-02036-8)
Supplement: Supplementary file 1 — Supplementary Information [file 41467_2017_2036_MOESM1_ESM.pdf]

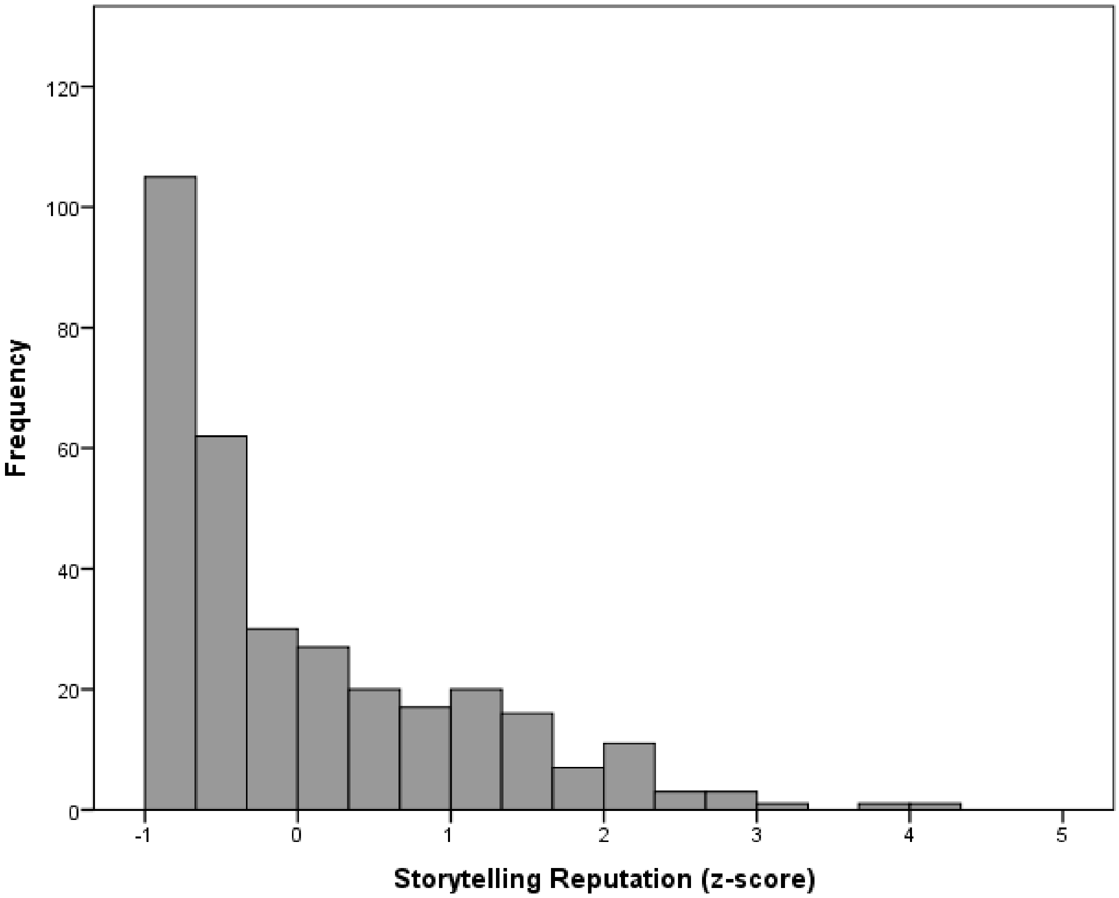

Supplementary Figure 1: Distribution of storytelling ability. There are few skilled (right of the figure), and many unskilled (left of the figure), storytellers (*n*=324). The number of nominations for individuals in each camp has been converted into *z*-scores to permit comparisons between camps of different sizes.

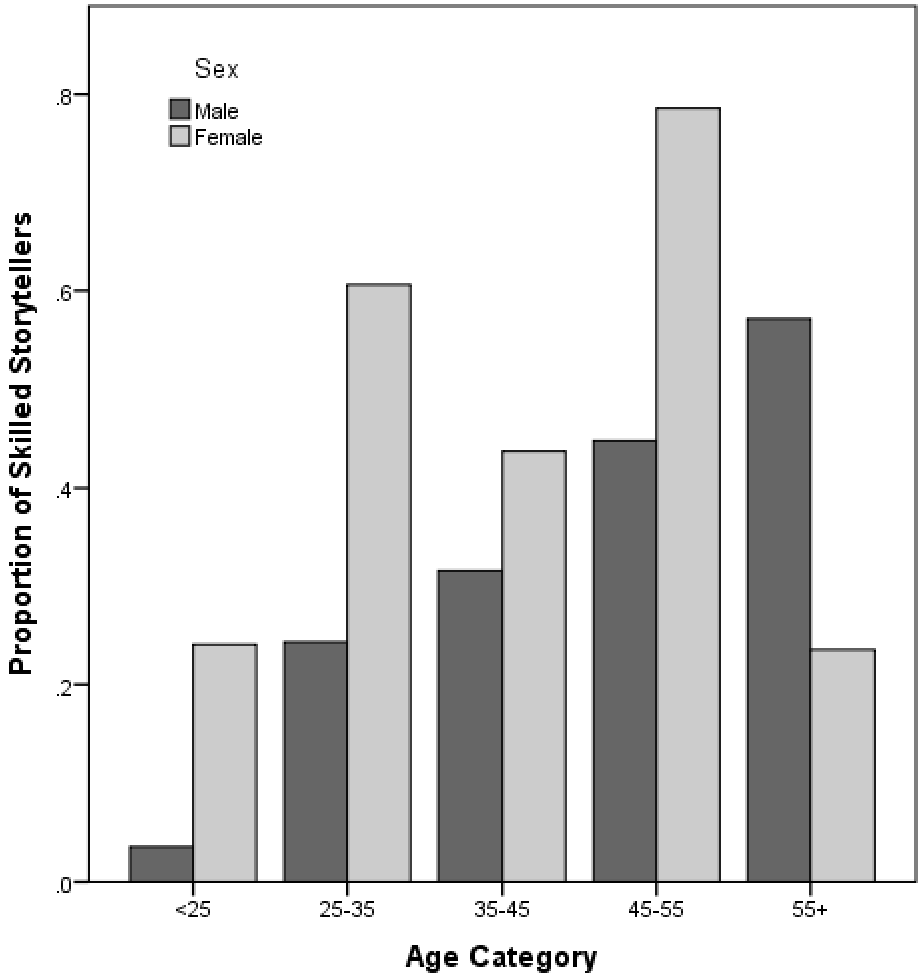

Supplementary Figure 2: Storytelling skill by age and sex. In a logistic regression containing age and sex, the probability of being a skilled storyteller increases with age for both sexes (*b*=0.04, 95% CI: [0.02; 0.06], $p<0.001$), but is more pronounced in males (*b*=0.04, 95% CI: [0.01; 0.08], $p=0.033$). For the purposes of this figure and to ease interpretation, ages have been categorised into discrete bins (*n*=324; males=dark grey, females=light grey).

Supplementary Table 1: Examples of stories concerning social behaviour from other Southeast Asian hunter-gatherer societies.

	**Andamanese (ref. [1])**	
**Story**	**Narrative**	**Promoted social norms**
Puluga and *Daria*	"Once upon a time *Puluga* and *Daria* [weather gods/spirits] were great friends, but they quarrelled. *Puluga* said that he was the bigger (more important). *Daria* said that she was. So now they are always quarrelling. *Puluga* sends the wind for one period. Then *Daria* sends his wind." (pg. 151)	Conflict avoidance
Creation	"When *Ta Peti* (Sir Monitor Lizard) was *aga-goi* (i.e., unmarried, but having completed the initiation ceremonies), he went into the jungle to hunt pig. He climbed up a *Diptercarpus* tree, and got stuck there. *Beyan* (civet-cat, *Paradoxurus*) found him there, stuck in the tree. She released him and helped him to get down. The two got married. Their children were the *Tomo-la* (i.e., the ancestors)." (pg. 193)	Cooperation between the sexes
Creation	"The first man was *Juptu*. He was born inside the joint of a big bamboo, just like a bird in an egg. The bamboo split and he came out. He was a little child. When it rained he made a small hut for himself and lived in it. He made little bows and arrows. As he grew bigger he made bigger huts, and bigger bows and arrows. One day he found a lump of quartz and with it scarified himself. *Juptu* was lonely, living all by himself. He took some clay (*kot*) from a nest of the white ants and moulded it into the shape of a woman. She became alive and became his wife. She was called *Kot*. They lived together at *Teraut-buliu*. Afterwards *Juptu* made other people out of clay. These were the ancestors. *Juptu* taught them how to make canoes and bows and arrows, and how to hunt and fish. His wife taught the women how to make baskets and nets and mats and belts, and how to use clay for making patterns on the body." (pg. 192)	Sexual division of labour
Bilika	"*Bilika* [thunder spirit] lived at *Poroy-et-co* with his wife *Mite*. They had a child. The ancestors ate *Bilika's* food, *loito* and *kata* and other plants. *Bilika* was very angry. He used to smell their mouths to see if they had eaten his food. When he found a man or woman who had done so he would cut his throat. The ancestors were very angry with *Bilika*, because he killed the men and women when they ate his foods. They all came together and killed *Bilika* and his wife *Mite*. *Maia Burto* (a species of fish) took the child (of *Bilika*) away to the north-east." (pg. 200)	Food sharing, punishment (killing) of non-sharers by peers
Puluga	"*Puluga* was always getting angry with the ancestors, because they dug up yams and ate *cakan* (*Entada scandens*) and *barata* (*Caryota sobolifera*). When he was angry he used to destroy the huts and property. So the people sent him out of the world, saying 'We do not want you here. You are always angry with us.' *Puluga* went away to the north-east." (pg. 200-201)	Punishment (ostracism, social exclusion) of violent people
Ta Mita and Ta Koio	"*Ta Mita* (dove) and *Ta Koio* (a small bird) went hunting together and got a great number of pigs. *Ta Koio* told *Ta Mita* to get some canes to tie up all the pigs. As soon as *Ta Mita* had gone to look for the cane, *Ta Koio* went up a big *Dipterocarpus* tree, taking half of the pigs with him. He came down and took the rest of the pigs. He	Food sharing, punishment (killing) of non-sharers by peers

	stayed up in the tree with the pigs. When *Ta Mita* came back he found that the pigs had disappeared. He was very angry and went home. As there was nothing to eat, *Mita* and his two children, *Cada* and *Coda* (two species of fish) went fishing. *Koio* was still up in the tree. He was cooking the pigs up there. *Mita* and his children passed under the tree and some burning resin fell on them. In this way they discovered that *Koio* was in the tree. *Mita* planned to punish *Koio*. He cut a great number of sharp stakes or Areca wood and fixed them all round the tree, pointing upwards. *Koio* was asleep. *Mita* made the tree sink into the ground. As soon as it was low enough he took some water and threw it onto the ear of the sleeping *Koio*, who awoke in a fright and jumped from his tree. He was impaled on the stakes of wood so died." (pg. 223-224)	
	**Batek (Malaysia: ref. [2])**	
Ya	"…two men got into a physical fight over one man's wife, whom both wanted. While a few people tried to break up the fight, most of the group fled the scene in panic, fearing that *Ya* [a deity] would split open the earth beneath the camp and destroy it in a massive flood. One man said he grabbed the wrists of the two combatants and said, "Think of the sun; think of the earth; this will all dissolve." (pg. 50)	Punishment (super-natural) of physical conflict
	**Maniq (Thailand: ref [3])**	
Selfish Maniq	"In the old days there was a group of Mani[q] who were very selfish. They did not share their food with others and just took the food found by other Maniq by force and ate it all, leaving nothing or very little. This causes hardship for the people in general. When the Maniq of the selfish group died, they were reincarnated as plants with leaves shaped like human fingers."	Food sharing, punishment (super-natural) of non-sharers.
	**Batak (Philippines: ref. [4])**	
Batak Padang	"Batak Padang … killed a large pig while hunting but left it to spoil in the forest, being too lazy, it was said, to carry such a heavy animal back to camp. About a year later, he was attacked and killed by a pig in the forest – a pig that was actually the *panya'en* [malevolent spirit], who had momentarily taken the animal's form." (pg. 10-11)	Punishment (super-natural) of laziness and greed
	**Aeta (Philippines: ref. [5])**	
Apo Alipon and Lola Moray	"The two Aetas Apo Alipon and Lola Moray settled in Morong, an area now called Barangay Mabayo. … Before Apo Alipon's death, he requested his descendents to dip their hands in the waters at the Kabuyaw river in Kanawan – a practice referred to as *kanaw* – and make an oath to the *anitos* (spirits) and Apo Namalyari (the pagan deity of happenings/events) that they will leave peacefully with one another and care for their land communally." (pg. 299)	Peaceful coexistence and common use of resources
	**Agta from Divilican (Philippines: ref. [6])**	
Creation	"A long time ago there were two birds. There was one male bird, his colour was black and his name was Uag, because he said 'uag-uag-uag'. The other bird was a female, white in colour, her name was Udok. Uag and Udok lived together in the skies. It was only them	Sex equality

and they were very lonely. Therefore they made a plan to create people to keep them company. In order to do so they came down from the skies, but they saw only a big blue ocean below and they needed something to land on. … They flew around to search for soil, from which they made flat land. While waiting for the soil to dry Uag and Udok realised that they should also make mountains. They put thick forest on the mountains and on the flat land. They created wild pigs, deer, birds, monkeys, fish, and other animals for the people to eat. Now it was time to put people on earth. Uag and Udok made love and Udok gave birth to a male and a female Agta. … These first Agta gave birth to five children, of which two again were an Agta couple and three were white. The three were a couple plus a male or female, who was adopted by the Agta. From then on, more and more people of all colours were born up to the present moment (pg. 71).”

Supplementary Table 2: Results of the story content analysis based on 89 stories obtained from seven hunter-gatherer societies.

Population (Number of Stories)	Social Content	Cosmological Content	Natural Phenomena	Resource Use
**Agta/Aeta (6)**	6 (100%)	2 (33%)	3 (50%)	1 (17%)
**Batak (2)**	2 (100%)	0 (0%)	0 (0%)	2 (100%)
**Maniq (8)**	2 (25%)	4 (50%)	4 (50%)	0 (0%)
**Batek (12)**	6 (50%)	8 (67%)	3 (25%)	2 (17%)
**Andamanese (41)**	25 (61%)	9 (22%)	23 (56%)	11 (27%)
**Ju/'hoansi (14)**	14 (100%)	0 (0%)	2 (14%)	12 (86%)
**BaYaka (6)**	6 (100%)	2 (33%)	0 (0%)	2 (33%)
**Total (89)**	61 (69%)	25 (28%)	35 (39%)	30 (34%)

Each story was assessed for four types of content: social, cosmological, natural phenomena and resource use. Each story could have two, or more, types of content (e.g., social and cosmological), hence why percentages add to over 100. The number of stories classified in each theme per society is displayed in cells with percentage of stories containing each type of content in brackets. Note that populations from northern Luzon in the Philippines (Agta and Aeta) have been grouped together here. Sources: **Agta/Aeta:** refs [5,6] and present study; **Batak:** ref. [4]; **Maniq**: ref. [3]; **Batek**: refs [2,7,8]; **Andamanese**: ref. [1]; **Ju/'hoansi**: ref s[9,10]; **BaYaka**: ref. [11].

Supplementary Table 3: Results of the multi-level model with percentage of resources kept for self in the resource allocation game as the dependent variable and average proportion of storytelling nominations per person in camp as the independent variable.

Variable	Parameter Estimate
**Average Proportion of Storytelling Nominations per Person in Camp**	-215.59 [-47.79; -388.37]*
**Camp Size**	-0.83 [-2.28; 0.62]
**Camp Relatedness**	-10.29 [-259.78; 239.18]
**Municipality (Ref. Maconacon)**	30.79 [12.27; 49.31]**
**Intercept**	67.24 [28.98; 105.5]**

Camp size, camp relatedness, and municipality are fixed-effect control variables, while camp membership is a group-level random effect. A higher parameter estimate indicates a greater proportion of resources kept for self. The negative estimate associated with proportion of storytelling nominations therefore indicates that individuals were more cooperative when there were a greater proportion of skilled storytellers in camp. 95% confidence intervals are displayed in brackets. *P*-value codes: ˙ <.1, * <.05, ** <.01, *** <.001.

Supplementary Table 4: Results of the multi-level model with percentage of resources kept for self in the resource allocation game as the dependent variable and average proportion of storytelling nominations per person in camp as the independent variable, controlling for the frequency of repeated interactions.

Variable	Parameter Estimate
**Average Proportion of Storytelling Nominations per Person in Camp**	-101.88 [-1.97; -201.78]*
**Camp Stability**	-78.01 [-56.43; -99.75]***
**Camp Size**	0.7 [-0.15; 1.55]
**Camp Relatedness**	-210.12 [-14.24; 434.48]·
**Intercept**	97.32 [75.51; 119.12]***

This model uses data from a sub-sample of 11 camps for which the frequency of repeated interactions could be assessed (*n*=183). This was possible as these 11 camps were visited multiple times, meaning that a metric of ‘camp stability’ could be derived, which ranged from ‘0’ (complete change in camp composition) to ‘1’ (no change in camp composition; for additional details, see ref. [12]). Controlling for camp stability (along with camp size and camp relatedness), individuals from camps with a greater proportion of skilled storytellers were still found to be significantly more cooperative, suggesting that this storytelling effect is unlikely to be due to increased familiarity between camp-mates. As with a previous publication[12], these results suggest that repeated interactions greatly facilitate cooperation (as stable camps were more cooperative), while the proportion of skilled storytellers possesses a smaller, although significant, positive independent effect. 95% confidence intervals are displayed in brackets. *P*-value codes: · <.1, * <.05, ** <.01, *** <.001.

Supplementary Table 5: Optimised model assessing the likelihood of skilled storytellers being selected in a 'camp-mate' network, using a logistic GEE regression (*n*=291, dyads=6,534).

**Variable**	**Model 4**	
	**Log-odds Ratio**	**Odds Ratio**
**Storytelling Reputation**	0.67 [0.49; 0.85]***	1.95
**Primary Kin (Ref. Non-kin)**	0.72 [0.33; 1.11]***	2.04
**Distant Kin (Ref. Non-kin)**	0.58 [0.29; 0.87]***	1.79
**Spouse's Primary Kin/ Primary Kin's Spouse (Ref. Non-kin)**	0.59 [0.26; 0.92]**	1.8
**Spouse's Distant Kin/Other Affines (Ref. Non-kin)**	0.24 [0; 0.48]˙	1.27
**Spouse (Ref. Non-kin)**	-0.29 [-1.01; 0.44]	0.75
**Reciprocity**	0.66 [0.46; 0.86]***	1.93
**Fishing Reputation**	0.57 [0.32; 0.82]***	1.76
**Age Gap**	-0.029 [-0.021; -0.037]***	0.97
**Ego Sex (Ref. Female)**	-0.34 [-0.16; -0.52]***	0.71
**Sex Diff (Ref. Same Sex)**	-2.38 [-2.03; -2.73]***	0.09
**Distance**	-0.34 [-0.22; -0.46]***	0.71
**Intercept**	0.79 [0.26; 1.32]**	-

This model includes only significant variables from model 3 (Table 2) in the main text. Removal of non-significant (or marginally significant (*p*>0.05)) variables from model 3 does not qualitatively alter the previous results, as individuals still overwhelmingly prefer to live with skilled storytellers. This model contains camp size as a control variable (not displayed). 95% confidence intervals are displayed in brackets. *P*-value codes: ˙ <.1, * <.05, ** <.01, *** <.001.

Supplementary Table 6: Multi-level models assessing the effects of storytelling ability on reproductive success (number of living offspring), controlling for age, age-squared, sex and camp (*n*=324).

Variable	Model 1: Binary, No Sex Control	Model 2: Continuous, No Sex Control	Model 3: Binary, Sex Control	Model 4: Continuous, Sex Control
**Age**	0.57 [0.49; 0.64]***	0.58 [0.48; 0.68]***	0.57 [0.49; 0.64]***	0.58 [0.5; 0.66]***
**Age-Squared**	-0.006 [-0.004; -0.008]***	-0.006 [-0.004; -0.008]***	-0.006 [-0.004; -0.008]***	-0.006 [-0.004; -0.008]***
**Sex**	-0.62 [-0.21; -1.03]**	-0.63 [-0.22; -1.04]**	-0.65 [-0.24; -1.06]**	-0.7 [-0.31; -1.09]***
**Storytelling Ability**	0.53 [0.1; 0.96]*	0.21 [-0.01; 0.43]˙	0.63 [0.2; 1.06]**	0.3 [0.08; 0.52]**
**Intercept**	-7.98 [-6.41; -9.55]***	-7.86 [-6.25; -9.47]***	-7.98 [-6.41; -9.55]***	-7.8 [-6.21; -9.39]***

Four models are displayed: Model 1 (with storytelling skill as a binary variable and no control for female-biased nominations of storytellers: this model is used in the main text); Model 2 (with storytelling skill a continuous variable and no control for female-biased nominations of storytellers); Model 3 (with storytelling skill a binary variable and female-biased nominations for storytellers controlled for); and Model 4 (with storytelling a continuous variable and female-biased nominations for storytellers controlled for). In each of these models, individuals with increased storytelling abilities possess greater reproductive success. 95% confidence intervals are displayed in brackets. *P*-value codes: ˙ <.1, * <.05, ** <.01, *** <.001.

Supplementary Table 7: Models assessing the likelihood of skilled storytellers (with storytelling as a continuous variable and no control for female-biased nominations of storytellers) being selected in a 'camp-mate' network, using a logistic GEE regression (*n*=291, dyads=6,534).

Variable	Model 1		Model 2		Model 3	
	Log-odds Estimate	Odds Ratio	Log-odds Estimate	Odds Ratio	Log-odds Estimate	Odds Ratio
**Storytelling Reputation**	0.24 [0.16; 0.32]***	1.27	0.33 [0.25; 0.41]***	1.4	0.26 [0.18; 0.34]***	1.29
**Primary Kin (Ref. Non-kin)**	-	-	0.76 [0.37; 1.15]***	2.14	0.7 [0..31; 1.09]***	2.01
**Distant Kin (Ref. Non-kin)**	-	-	0.63 [0.34; 0.92]***	1.87	0.61 [0.21; 0.9]***	1.83
**Spouse's Primary Kin/ Primary Kin's Spouse (Ref. Non-kin)**	-	-	0.61 [0.26; 0.96]***	1.85	0.57 [0.21; 0.92]**	1.77
**Spouse's Distant Kin/Other Affines (Ref. Non-kin)**	-	-	0.29 [0.04; 0.54]*	1.34	0.27 [0.02; 0.52]*	1.31
**Spouse (Ref. Non-kin)**	-	-	-0.23 [-0.96; 0.5]	0.8	-0.29 [-1.03; 0.45]	0.75
**Reciprocity**	-	-	0.67 [0.47; 0.87]***	1.95	0.66 [0.46; 0.86]***	1.94
**Fishing Reputation**	-	-	-	-	0.22 [0.12; 0.32]***	1.24
**Hunting Reputation**	-	-	-	-	0.04 [-0.06; 0.14]	1.04
**Tuber Gathering Reputation**	-	-	-	-	0.2 [0.06; 0.34]**	1.22
**Medicinal Knowledge Reputation**	-	-	-	-	-0.01 [-0.11; 0.09]	0.99
**Camp Influence Reputation**	-	-	-	-	0.15 [0.07; 0.23]***	1.16
**Intercept**	-0.62 [-0.44; -0.8]***		1.19 [0.62; 1.76]***		1.54 [0.95; 2.13]***	
**Distance, Age, and Sex Controls**	No		Yes		Yes	

All models contain camp size as a control variable (not displayed). For consistency with storytelling skill, other reputational domains have also been inputted as continuous variables. 95% confidence intervals are displayed in brackets. *P*-value codes: ˙ <.1, * <.05, ** <.01, *** <.001.

Supplementary Table 8: Models assessing the likelihood of skilled storytellers (with storytelling as a binary variable and female-biased nominations of storytellers controlled for) being selected in a ‘camp-mate’ network, using a logistic GEE regression (*n*=291, dyads=6,534).

Variable	Model 1		Model 2		Model 3	
	Log-odds Estimate	Odds Ratio	Log-odds Estimate	Odds Ratio	Log-odds Estimate	Odds Ratio
**Storytelling Reputation**	0.54 [0.38; 0.7]***	1.71	0.7 [0.5; 0.9]***	2.01	0.58 [0.38; 0.78]***	1.78
**Primary Kin (Ref. Non-kin)**	-	-	0.73 [0.34; 1.12]***	2.08	0.69 [0.3; 1.08]***	2
**Distant Kin (Ref. Non-kin)**	-	-	0.59 [0..3; 0.88]***	1.8	0.57 [0.28; 0.86]***	1.77
**Spouse’s Primary Kin/ Primary Kin’s Spouse (Ref. Non-kin)**	-	-	0.61 [0.28; 0.94]***	1.84	0.58 [0.25; 0.91]***	1.79
**Spouse’s Distant Kin/Other Affines (Ref. Non-kin)**	-	-	0.26 [0.01; 0.51]*	1.29	0.24 [-0.01; 0.49]˙	1.28
**Spouse (Ref. Non-kin)**	-	-	-0.24 [-0.97; 0.49]	0.78	-0.27 [-1; 0.46]	0.76
**Reciprocity**	-	-	0.65 [0.45; 0.85]***	1.92	0.66 [0.46; 0.86]***	1.93
**Fishing Reputation**	-	-	-	-	0.39 [0.12; 0.66]**	1.48
**Hunting Reputation**	-	-	-	-	0.2 [-0.07; 0.47]	1.22
**Tuber Gathering Reputation**	-	-	-	-	0.32 [0.05; 0.59]*	1.38
**Medicinal Knowledge Reputation**	-	-	-	-	0.1 [-0.14; 0.34]	1.1
**Camp Influence Reputation**	-	-	-	-	0.28 [0.03; 0.53]*	1.33
**Intercept**	-0.85 [-0.65; -1.05]***		0.94 [0.37; 1.51]**		0.97 [0.38; 1.56]**	
**Distance, Age, and Sex Controls**	No		Yes		Yes	

All models contain camp size as a control variable (not displayed). 95% confidence intervals are displayed in brackets. *P*-value codes: ˙ <.1, * <.05, ** <.01, *** <.001.

Supplementary Table 9: Models assessing the likelihood of skilled storytellers (with storytelling as a continuous variable and female-biased nominations of storytellers controlled for) being selected in a 'camp-mate' network, using a logistic GEE regression (*n*=291, dyads=6,534).

Variable	Model 1		Model 2		Model 3	
	Log-odds Estimate	Odds Ratio	Log-odds Estimate	Odds Ratio	Log-odds Estimate	Odds Ratio
**Storytelling Reputation**	0.25 [0.19; 0.31]***	1.29	0.34 [0.26; 0.42]***	1.4	0.24 [0.14; 0.34]***	1.28
**Primary Kin (Ref. Non-kin)**	-	-	0.74 [0.35; 1.13]***	2.1	0.69 [0.3; 1.08]***	1.99
**Distant Kin (Ref. Non-kin)**	-	-	0.62 [0.33; 0.91]***	1.86	0.61 [0.32; 0.9]***	1.83
**Spouse's Primary Kin/ Primary Kin's Spouse (Ref. Non-kin)**	-	-	0.61 [0.28; 0.94]***	1.84	0.57 [0.24; 0.9]**	1.77
**Spouse's Distant Kin/Other Affines (Ref. Non-kin)**	-	-	0.28 [0.03; 0.53]*	1.32	0.26 [0.01; 0.51]*	1.29
**Spouse (Ref. Non-kin)**	-	-	-0.25 [-0.98; 0.48]	0.78	-0.3 [-1.04; 0.44]	0.74
**Reciprocity**	-	-	0.66 [0.46; 0.86]***	1.93	0.65 [0.45; 0.85]***	1.92
**Fishing Reputation**	-	-	-	-	0.23 [0.13; 0.33]***	1.25
**Hunting Reputation**	-	-	-	-	0.02 [-0.09; 0.12]	1.02
**Tuber Gathering Reputation**	-	-	-	-	0.17 [0.05; 0.29]**	1.19
**Medicinal Knowledge Reputation**	-	-	-	-	0.01 [-0.09; 0.11]	1.01
**Camp Influence Reputation**	-	-	-	-	0.14 [0.06; 0.22]***	1.16
**Intercept**	-0.62 [-0.44; -0.8]***		1.24 [0.67; 1.81]***		1.53 [0.94; 2.12]***	
**Distance, Age, and Sex Controls**	No		Yes		Yes	

All models contain camp size as a control variable (not displayed). For consistency with storytelling skill, other reputational domains have also been inputted as continuous variables. 95% confidence intervals are displayed in brackets. *P*-value codes: ˙ <.1, * <.05, ** <.01, *** <.001.

Supplementary Table 10: Models assessing the likelihood of skilled storytellers being selected to receive resources in the experimental game, using a logistic GEE regression (*n*=290, dyads=1,312).

Variable	Model 1		Model 2		Model 3	
	Log-odds Estimate	Odds Ratio	Log-odds Estimate	Odds Ratio	Log-odds Estimate	Odds Ratio
**Storytelling Reputation**	0.38 [0.09; 0.68]*	1.46	0.42 [0.09; 0.76]*	1.53	0.37 [0.03; 0.72]*	1.45
**Primary Kin (Ref. Non-kin)**	-	-	1.33 [0.7; 1.96]***	3.8	1.33 [0.7; 1.96]***	3.78
**Distant Kin (Ref. Non-kin)**	-	-	0.6 [0.03; 1.18]*	1.83	0.61 [0.03; 1.19]*	1.84
**Spouse's Primary Kin/ Primary Kin's Spouse (Ref. Non-kin)**	-	-	0.18 [-0.42; 0.79]	1.2	0.16 [-0.46; 0.78]	1.17
**Spouse's Distant Kin/Other Affines (Ref. Non-kin)**	-	-	0.04 [-0.4; 0.49]	1.04	0.05 [-0.4; 0.5]	1.05
**Spouse (Ref. Non-kin)**	-	-	0.01 [-0.91; 0.93]	1.01	-0.01 [-0.93; 0.92]	0.99
**Reciprocity**	-	-	0.33 [0.02; 0.63]*	1.39	0.31 [0; 0.62]*	1.37
**Fishing Reputation**	-	-	-	-	0.26 [-0.21; 0.72]	1.29
**Hunting Reputation**	-	-	-	-	0.27 [-0.23; 0.77]	1.32
**Tuber Gathering Reputation**	-	-	-	-	0.13 [-0.41; 0.68]	1.14
**Medicinal Knowledge Reputation**	-	-	-	-	-0.03 [-0.46; 0.41]	0.97
**Camp Influence Reputation**	-	-	-	-	0.19 [-0.29; 0.67]	1.21
**Intercept**	2.69 [2.35; 3.03]***		2.58 [1.63; 3.52]***		2.64 [1.69; 3.59]***	
**Distance, Age, and Sex Controls**	No		Yes		Yes	

As with the 'camp-mate' network (Table 2), three models are displayed: Model 1, a univariate model between resource distributions and storytelling reputation; Model 2, which contains additional controls for kinship, reciprocity, distance, as well as age and sex variables; and Model 3, which also included assessments of reputation in other domains. An optimised model containing just significantly predictive variables from model 3 is displayed in Supplementary Table 10. In all models skilled storytellers were significantly more likely to receive resources, with odds ratios indicating that they were approximately 50% more likely to receive resources compared to non-skilled storytellers. All models contain number of resources distributed as a control variable to ensure that patterns of resource allocation were not confounded with overall levels of cooperativeness (not displayed). 95% confidence intervals are displayed in brackets. *P*-value codes: · <.1, * <.05, ** <.01, *** <.001.

Supplementary Table 11: Optimised model assessing the likelihood of skilled storytellers being selected to receive resources in the experimental game, using a logistic GEE regression (*n*=290, dyads=1,312).

**Variable**	**Model 4**	
	**Log-odds Ratio**	**Odds Ratio**
**Storytelling Reputation**	0.5 [0.19; 0.81]**	1.65
**Primary Kin (Ref. Non-kin)**	1.43 [0.86; 1.99]***	4.16
**Distant Kin (Ref. Non-kin)**	0.63 [0.09; 1.18]*	1.88
**Spouse's Primary Kin/ Primary Kin's Spouse (Ref. Non-kin)**	0.27 [-0.3; 0.84]	1.31
**Spouse's Distant Kin/Other Affines (Ref. Non-kin)**	0.08 [-0.32; 0.49]	1.09
**Spouse (Ref. Non-kin)**	0.11 [-0.62; 0.84]	1.11
**Reciprocity**	0.34 [0.04; 0.65]*	1.41
**Sex Diff (Ref. Same Sex)**	-0.65 [-0.29; -1.01]***	0.52
**Intercept**	2.57 [2.03; 3.11]***	-

This model includes only significant variables from model 3 in Supplementary Table 10. Removal of non-significant variables from this model does not qualitatively alter the previous results, as individuals still prefer to share resources with skilled storytellers. This model contains number of resources distributed as a control variable to ensure that patterns of resource allocation were not confounded with overall levels of cooperativeness (not displayed). 95% confidence intervals are displayed in brackets. *P*-value codes: $^{\bullet}$ <.1, * <.05, ** <.01, *** <.001.

Supplementary Table 12: Models assessing the likelihood of skilled storytellers being selected in a 'camp-mate' network (*n*=291, dyads=6,534) or to receive resources in the experimental game (*n*=290, dyads=1,312) with an 'overall foraging skill' variable replacing sex-specific foraging skills (hunting (male-biased), fishing (male-biased) and tuber-gathering (female-biased)).

Variable	Camp-Mate Model		Experimental Game Model	
	Log-odds Estimate	Odds Ratio	Log-odds Estimate	Odds Ratio
**Storytelling Reputation**	0.67 [0.47; 0.87]***	1.95	0.37 [0.03; 0.71]*	1.45
**Primary Kin (Ref. Non-kin)**	0.71 [0.32; 1.1]***	2.03	1.35 [0.71; 1.98]***	3.84
**Distant Kin (Ref. Non-kin)**	0.59 [0.29; 0.88]***	1.8	0.61 [0.03; 1.2]*	1.85
**Spouse's Primary Kin/ Primary Kin's Spouse (Ref. Non-kin)**	0.58 [0.23; 0.92]***	1.78	0.17 [-0.44; 0.78]	1.18
**Spouse's Distant Kin/Other Affines (Ref. Non-kin)**	0.25 [0.0; 0.49]*	1.28	0.05 [-0.41; 0.51]	1.05
**Spouse (Ref. Non-kin)**	-0.27 [-1; 0.46]	0.77	0.0 [-0.92; 0.93]	1
**Reciprocity**	0.67 [0.47; 0.87]***	1.95	0.32 [0.01; 0.63]*	1.37
**Overall Foraging Reputation**	0.32 [0.13; 0.51]***	1.37	0.2 [-0.13; 0.71]	1.22
**Medicinal Knowledge Reputation**	0.09 [-0.14; 0.32]	1.09	-0.08 [-0.49; 0.34]	0.93
**Camp Influence Reputation**	0.23 [-0.02; 0.48] ˙	1.25	0.2 [-0.25; 0.66]	1.22
**Intercept**	0.93 [0.36; 1.49]***		2.58 [1.63; 3.52]***	
**Distance, Age, and Sex Controls**	Yes		Yes	

As with Model 3 in the respective tables (Table 2 for the camp-mate network and Supplementary Table 10 for the resource allocation game network), this analysis contains the full list of possible predictor variables. Models utilise logistic GEE regressions with camp size as a control variable for the camp-mate network and number of resources distributed as a control variable for the experimental game network (both not displayed). 95% confidence intervals are displayed in brackets. *P*-value codes: ˙ <.1, * <.05, ** <.01, *** <.001.

Supplementary Table 13: Correlation matrix displaying the association between each of the reputational domains (*n*=324).

	Hunting Reputation	Fishing Reputation	Tuber-Gathering Reputation	Story-telling Reputation	Medicinal Knowledge Reputation	Camp Influence Reputation
**Hunting Reputation**	-	0.274***	-0.241***	0.06	-0.05	0.254***
**Fishing Reputation**	-	-	-0.256***	-0.011	-0.181***	0.221***
**Tuber-Gathering Reputation**	-	-	-	0.292***	-0.401***	-0.084
**Storytelling Reputation**	-	-	-	-	0.3***	0.276***
**Medicinal Knowledge Reputation**	-	-	-	-	-	0.255***
**Camp Influence Reputation**	-	-	-	-	-	-

Although some correlations are significant, the effect sizes are relatively weak (all bar one are $r \leq 0.3$). Reputation is measured as a binary variable, with '1' indicating skill in said domain (a *z*-score above '0'), and less-skilled individuals given a '0' (a *z*-score lower than '0'). Note that each of these domains includes both males and females, even for sex-specific tasks such as hunting, fishing, and tuber-gathering. *P*-value code: *** <.001.

Supplementary References

1. Radcliffe-Brown, A. *The Andaman Islanders: A Study in Social Anthropology*. (Cambridge University Press, 1922).
2. Endicott, K. & Endicott, K. L. *The Headman was a Woman: The Gender Egalitarian Batek of Malaysia*. (Waveland Press, 2008).
3. Thonghom, S. The Negrito of Thailand: The Mani. (2006). Available at: http://web.archive.org/web/20130520173144/http://www.andaman.org/BOOK/chapter36/text36.htm. (Accessed: 26th May 2017)
4. Eder, J. *Batak Resource Management: Belief, Knowledge and Practice*. (IUCN and WWF, 1997).
5. McHenry, M. P., Anwar McHenry, J., Balilla, V. S. & Parkinson, R. M. The Indigenous Aetas of Bataan, Philippines: Extraordinary Genetic Origins, Modern History and Land Rights. *Singap. J. Trop. Geogr.* **34,** 292–306 (2013).
6. Minter, T. The Agta of the Northern Sierra Madre: Livelihood Strategies and Resilience among Philippine Hunter-Gatherers. (Leiden University, 2010).
7. Endicott, K. *Batek Negrito Religion: The World-View and Rituals of a Hunting and Gathering People of Peninsular Malaysia*. (Claredon Press, 1979).
8. Lye, T.-P. *Changing Pathways: Forest Degradation and the Batek of Pahang, Malaysia*. (Lexington Books, 2005).
9. Biesele, M. *Women Like Meat: The Folklore and Foraging Ideology of the Kalahari Ju/'hoan*. (Indiana University Press, 1993).
10. Wiessner, P. Embers of society: Firelight talk among the Ju/'hoansi Bushmen. *Proc. Natl. Acad. Sci.* **111,** 14027–14035 (2014).
11. Lewis, J. Forest hunter-gatherers and their world: a study of the Mbendjele Yaka pygmies of Congo-Brazzaville and their secular and religious activities and representations. (London School of Economics and Political Science, 2002).
12. Smith, D. *et al.* Camp stability predicts patterns of hunter-gatherer cooperation. *R. Soc. Open Sci.* **3,** 160131 (2016).
